# The role of plectin as a druggable target in papillary thyroid carcinoma: therapeutic potential of troglitazone

**DOI:** 10.3389/fimmu.2026.1789251

**Published:** 2026-08-26

**Authors:** Yi-Chen Lv, Jie-Hao Zhou, Tie-Feng Shi, Yun-Fu Cui

**Affiliations:** 1 Department of Pancreatobiliary Surgery, The Second Affiliated Hospital of Harbin Medical University, Harbin, Heilongjiang, China; 2 Departments of General Surgery, The Second Affiliated Hospital of Harbin Medical University, Harbin, Heilongjiang, China; 3 Departments of Digestive Diagnosis and Treatment, First Affiliated Hospital of Heilongjiang University of Chinese Medicine, Harbin, Heilongjiang, China

**Keywords:** molecular docking, molecular subtyping, papillary thyroid carcinoma, plectin, troglitazone

## Abstract

**Objective:**

This study aimed to characterize molecular subtypes of Papillary thyroid carcinoma (PTC) and identify novel therapeutic targets and potential therapeutic compounds.

**Methods:**

We performed integrative analysis of TCGA data, including consensus clustering, mutational profiling, copy number variation (CNV) analysis, functional enrichment (GO and GSEA), and immune microenvironment assessment (CIBERSORT). Validation utilized GEO datasets. The biological function of Plectin (PLEC) was investigated through expression analysis (TCGA, GEO, qRT-PCR and IHC), association with clinical parameters, and functional experiments such as siRNA knockdown or overexpression of PLEC in TPC-1 and B-CPAP cell lines assessing cell growth, proliferation, apoptosis, and migration both *in vitro* and *in vivo*. Drug candidates targeting PLEC were screened (DGIdb and DrugMap) and validated via molecular docking, dynamics simulations (RMSD, RMSF and Rg), and rescue experiments. Favorable biosafety was tested by ELISA kits and H&E staining.

**Results:**

Consensus clustering revealed four stable molecular subtypes (A1-A4) with distinct mutational landscapes (e.g., prevalent *BRAF* mutations (59%), subtype-specific alterations in *CAND1/PCDH11X* (A1) and *PTEN* (A4)), CNV gains (e.g., A1: 2p16.1/19p13.3), and immune features (A3 enriched in immune pathways). PLEC is up-regulated in PTC tissues compared with non-tumor thyroid tissues and was associated with lymph node metastasis, histological type, and residual tumor status in TCGA-THCA. Knockdown of PLEC significantly suppressed PTC cell growth, proliferation, and migration while inducing apoptosis. Whereas PLEC overexpression partially reversed these phenotypes. Among FDA-approved drugs predicted to target PLEC, Troglitazone exhibited the lowest binding affinity (-7.5 kcal/mol). Troglitazone’s anti-tumor effects (inhibiting proliferation/migration, inducing apoptosis) were partially reversed by PLEC overexpression, suggesting that PLEC may participate in Troglitazone-associated anti-PTC effects.

**Conclusion:**

These findings identify PLEC as a candidate molecule associated with aggressive PTC phenotypes and provide preliminary evidence supporting its biological relevance, although larger independent cohorts are required to validate its clinical significance.

## Introduction

Papillary thyroid carcinoma accounts for 80-90% of thyroid malignancies, with increasing global incidence driven primarily by improved diagnostic techniques (1–3). Although most patients experience favorable outcomes (5-year survival >95%), 10-30% develop aggressive disease characterized by lymph node metastasis, iodine refractoriness, and distant dissemination-factors contributing significantly to recurrence and mortality in high-risk subgroups (4, 5). Genomic profiling has established BRAF/V600E (∼60% prevalence) and RAS mutations as key drivers of MAPK signaling activation (6–8), which is closely related to tumorigenesis (9, 10). However, their limited specificity—BRAF/V600E/occurs in 40-60% of melanomas and 5-15% of colorectal cancers—and absence in ∼50% of PTC cases highlight the critical need for subtype-specific diagnostic biomarkers (11). Molecular heterogeneity remains inadequately mapped to actionable therapeutic vulnerabilities, particularly in advanced cases resistant to conventional therapies.

The *PLEC* gene encodes plectin, a large cytolinker protein that connects intermediate filaments with actin microfilaments, microtubules, hemidesmosomes, and focal adhesion-related structures (12, 13). Through these functions, plectin contributes to cytoskeletal organization, mechanical signal transduction, cell adhesion, and tissue integrity (14, 15). In epithelial cancers, aberrant plectin expression has been associated with tumor migration, metastatic dissemination, altered cell-matrix interaction, and treatment resistance, including reported roles in pancreatic, ovarian, esophageal, and hepatocellular carcinoma models (16, 17). These findings suggest that plectin may participate in epithelial tumor progression, but its expression pattern, clinical relevance, and functional contribution in PTC remain insufficiently characterized.

This study addresses this gap through an integrated analytical and experimental framework. First, we performed molecular subtype analysis using public thyroid cancer transcriptomic and genomic datasets. Second, we evaluated the expression and preliminary clinical relevance of *PLEC* in PTC using TCGA, GEO datasets, and paired clinical samples. Third, proliferation, invasion, and apoptosis are key events in tumor biology, therefore we tested the impact of PLEC on these progresses *in vitro* and *in vivo (*18–22). Finally, we explored candidate compounds associated with PLEC using drug-gene interaction resources, molecular docking, molecular dynamics simulation, CETSA, and functional assays. Together, these analyses were designed to clarify whether PLEC may represent a biologically relevant candidate molecule in PTC, while avoiding direct claims of clinical utility or immediate therapeutic translation.

## 2 Materials and methods

### 2.1 Data acquisition, preprocessing, and dataset harmonization

Publicly available thyroid cancer datasets were obtained from The Cancer Genome Atlas (TCGA) through the Genomic Data Commons (GDC) portal and from the Gene Expression Omnibus (GEO). TCGA-THCA data were downloaded on Oct 1, 2025, using the following filters: project, TCGA-THCA; primary site, thyroid gland; disease type, adenomas and adenocarcinomas; and access category, open. The downloaded TCGA data comprised gene-level RNA-seq STAR-Counts files (n = 551), masked somatic mutation files in mutation annotation format (MAF; n = 483), and masked copy-number segment files (n = 991). Gene-level count files rather than raw FASTQ sequencing reads were analyzed. Clinicopathological association analyses included 512 patients with PTC for whom the required clinical annotations were available.

For subtype-related external validation, GSE104005 was obtained from GEO. GSE104005 was generated using the Illumina HumanHT-12 WG-DASL V4.0 R2 expression beadchip (GPL14951) and includes different thyroid carcinoma histotypes and non-neoplastic thyroid tissues. The present analysis retained 29 thyroid carcinoma samples and 5 non-neoplastic thyroid samples. Because the carcinoma group included PTC, poorly differentiated thyroid carcinoma, and lymph-node metastasis-derived samples, GSE104005 was treated as an external thyroid carcinoma cohort rather than a PTC-only cohort.

PLEC expression was additionally evaluated in GSE33630 and GSE60542. Both datasets were generated using the Affymetrix Human Genome U133 Plus 2.0 Array (GPL570). For GSE33630, 49 PTC samples and 45 normal thyroid samples were retained after excluding anaplastic thyroid carcinoma samples. For GSE60542, 24 primary PTC samples and 11 normal thyroid samples were retained after excluding lymph-node metastases, normal lymph nodes, recurrent or distant metastatic lesions, and technical or biological replicates not used in the present comparison. The accession number, platform, full cohort composition, retained sample number, input-data type, and analytical purpose of each dataset are summarized in **Supplementary Table 1**.

### 2.2 Molecular subtype classification

Molecular subtypes were determined by unsupervised consensus clustering using the R package “Consensus Cluster Plus” (v1.60.0) based on gene expression profiles. Based on these criteria, k = 4 was selected, defining the A1–A4 molecular subtypes. The resulting subtype assignments were used in subsequent mutation, copy-number, pathway, immune-infiltration, and clinicopathological analyses.

### 2.3 Genomic alteration profiling

Somatic mutation data in MAF format from TCGA-THCA were analyzed using maftools (v2.16.0). Mutation landscapes were summarized for the overall cohort and for each molecular subtype. Copy number variation data were analyzed using GISTIC 2.0 based on TCGA masked copy number segment files. SNP6 GRCh38 remapping reference files from the GDC reference resource were used for CNV preprocessing when applicable. Recurrent amplification and deletion regions were identified for each subtype and used for downstream functional interpretation.

### 2.4 Functional enrichment analysis

Differential expression analysis for GEO microarray datasets was performed using the limma workflow after probe annotation and expression normalization. Differentially expressed genes were defined as genes with |log2 fold change| > 1 and Benjamini-Hochberg adjusted *P* < 0.05. When multiple probes mapped to the same gene, the probe with the highest expression value was retained unless otherwise specified. GO enrichment analysis was performed using clusterProfiler (v4.6.2), and adjusted *P* values were calculated using the Benjamini-Hochberg method. Hallmark pathway activity was evaluated using GSEA (v1.48.2) based on MSigDB Hallmark gene sets. Enrichment or pathway terms with adjusted P < 0.05 were considered statistically significant.

### 2.5 Tumor immune microenvironment analysis

Immune-cell composition was estimated from the normalized TCGA-THCA RNA-seq expression matrix using the CIBERSORT R script obtained from the official CIBERSORT website on (Oct/1/2025), together with the LM22 leukocyte gene-signature matrix. Quantile normalization was enabled according to the actual analysis settings. The estimated fractions of the 22 immune-cell populations were compared across the A1–A4 molecular subtypes using Wilcoxon rank-sum test. Where applicable, P values were adjusted for multiple comparisons using the Benjamini–Hochberg method, and *P* <0.05 was considered statistically significant.

### 2.6 ROC analysis

**Figure 1:**
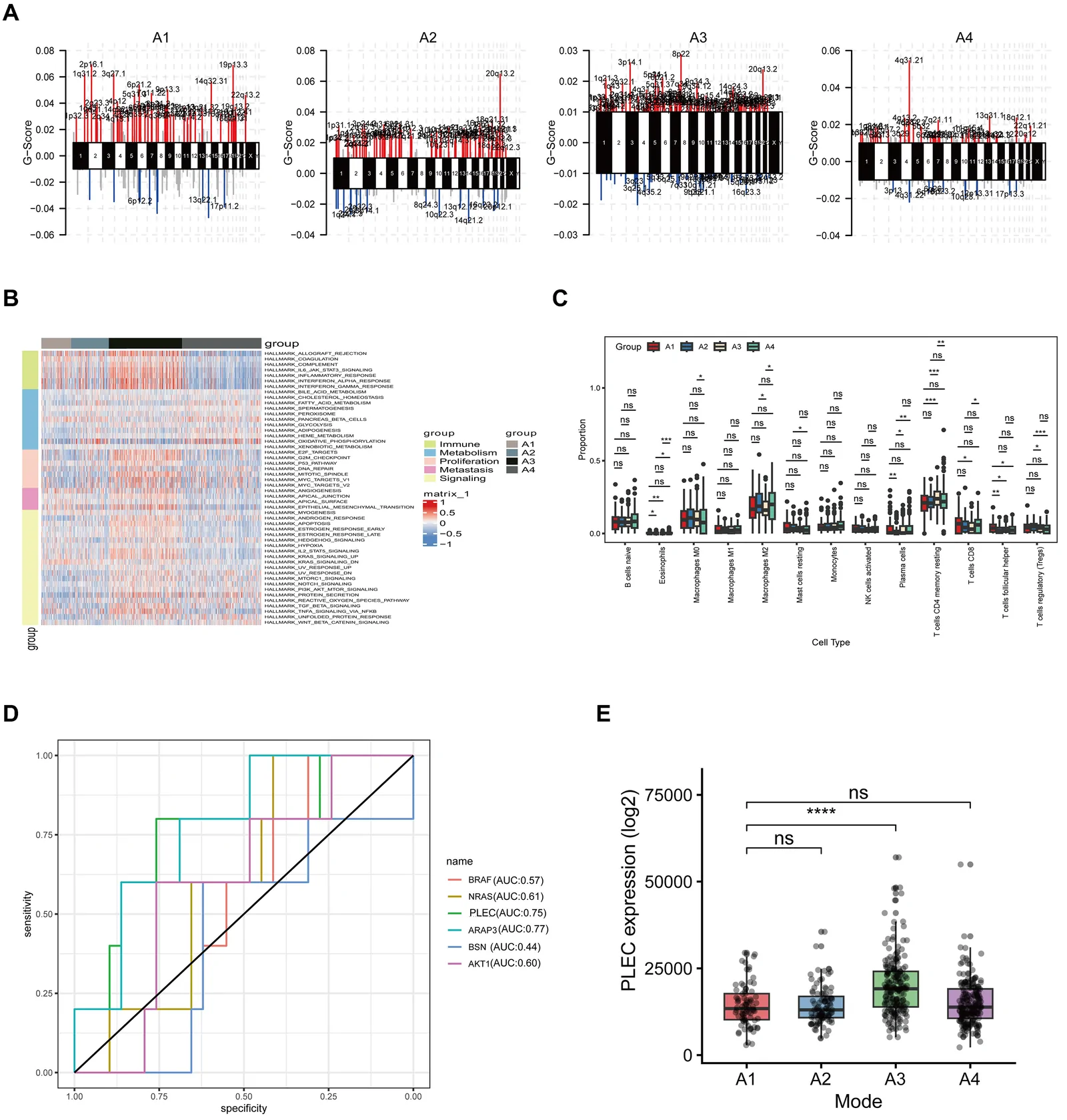
Functional enrichment and immune microenvironment characteristics of molecular subtypes. **(A)** Copy number variation profiles and representative enriched GO terms for each subtype. Red indicates copy number gain, and blue indicates copy number loss. Bubble size represents gene count, and color indicates adjusted P value. **(B)** Hallmark pathway activity estimated by GSEA across molecular subtypes. **(C)** Immune cell infiltration fractions estimated by CIBERSORT across A1-A4 subtypes. Statistical analysis was performed using Wilcoxon rank-sum test, with multiple-comparison correction where applicable. **(D)** ROC analysis of subtype-associated markers in the GEO validation cohort GSE104005. AUC values and 95% confidence intervals are shown. **(E)** The expression of PLEC across the A1-A4 subtypes. ***P < 0.001, nsP > 0.05.

Receiver operating characteristic (ROC) analysis was performed using the R package pROC (v1.18.4). For **Figure 1D**, ROC analysis was conducted using the external GEO validation dataset GSE104005 to evaluate the discriminatory performance of A4-associated mutation-related molecular markers between thyroid carcinoma and non-neoplastic thyroid tissues. After applying the inclusion criteria used in the present analysis, GSE104005 included 29 thyroid carcinoma samples and 5 non-neoplastic thyroid samples. Because this GEO cohort contained mixed thyroid carcinoma histological types, this analysis was interpreted as thyroid carcinoma versus non-neoplastic thyroid discrimination rather than a PTC-only validation.

**Figure 2:**
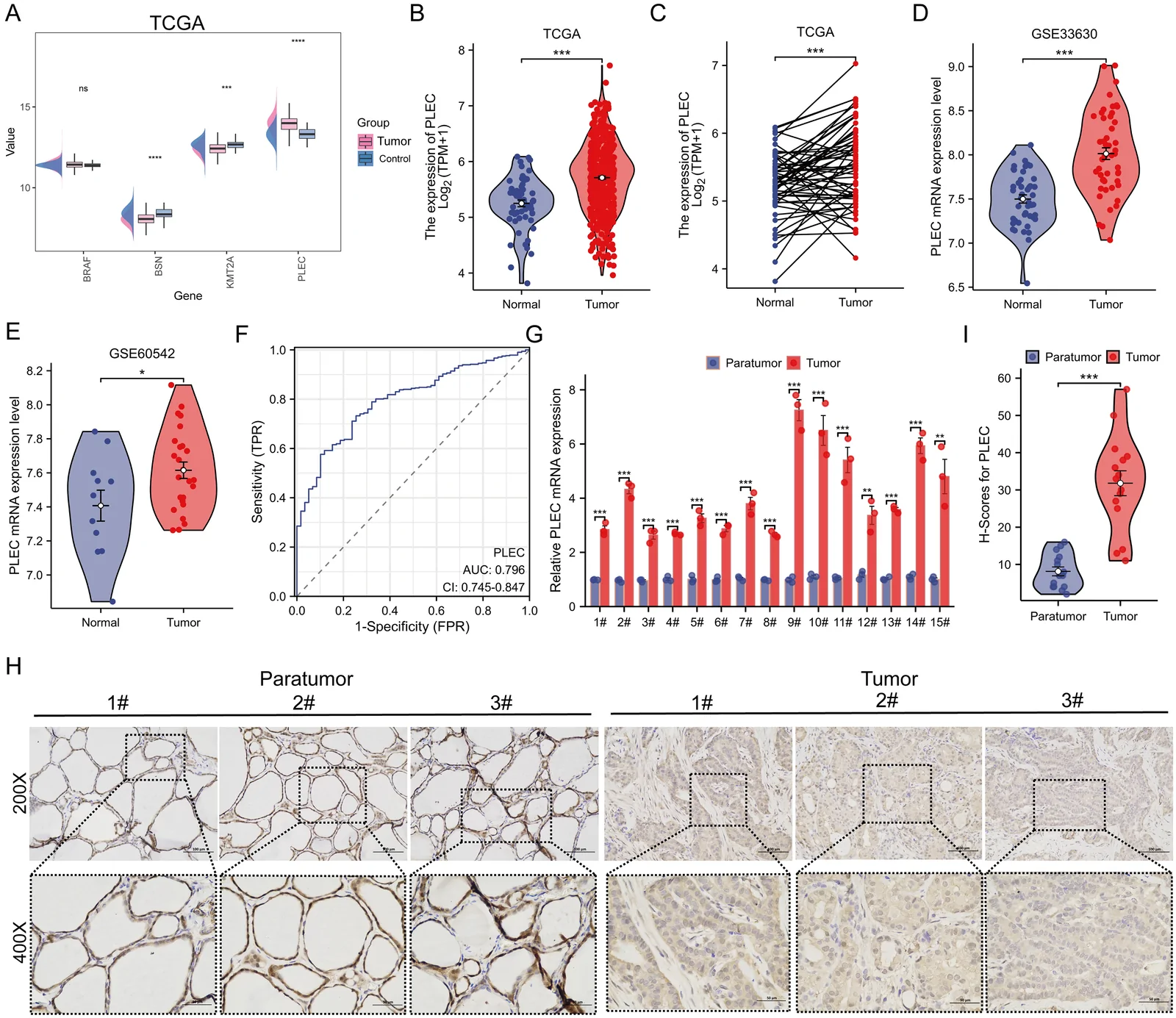
PLEC is upregulated in PTC and shows preliminary diagnostic relevance. **(A–C)** Differential expression analysis of hub genes, including *PLEC*, in PTC and non-tumor thyroid tissues from TCGA. **(D, E)** Validation of *PLEC* expression in independent GEO datasets GSE33630 and GSE60542. **(F)** ROC analysis evaluating the preliminary diagnostic relevance of *PLEC*. **(G)** RT-qPCR analysis of *PLEC* mRNA expression in 15 paired PTC and adjacent non-tumorous tissues. **(H, I)** Representative immunohistochemical staining and H-score quantification of PLEC protein in 15 paired PTC and adjacent non-tumorous tissues. Data are presented as the mean ± SD. Statistical analysis was performed using Student’s t-test. *P < 0.05; **P < 0.01; ***P < 0.001.

For **Figure 2F**, ROC analysis was performed using the TCGA-THCA cohort to evaluate the ability of PLEC expression to distinguish PTC tissues from non-tumor thyroid tissues. According to the TCGA-based analysis used for **Figure 2**, 512 PTC samples and 59 non-tumor thyroid samples were included. AUC values were calculated to estimate discriminatory performance, and 95% confidence intervals were estimated using DeLong’s method when applicable. The exact dataset source, platform or data type, sample size, marker, grouping definition, and purpose of each ROC analysis are summarized in **Supplementary Table 2**.

### 2.7 Reagents and antibodies

Troglitazone (CAS 97322-87-7; purity ≥96%) was obtained from Aladdin Biotechnology Co., Ltd. (Shanghai, China). The primary antibody against PLEC (#R380761) was purchased from Zenbio (Chengdu, China). The β-actin antibody (#GB15001-100), TRIzol™ reagent (#G3013), 10% SDS–PAGE gels (#G2178-50T), BCA protein assay kits (#G2026), and West ECL substrates (#G2014) were purchased from Servicebio (Wuhan, China). Nitrocellulose membranes(#NC10600001) were obtained from Cytiva (USA). Assay kits for ALT (#C009-2-1), AST (#C010-2-1), CRE (#C011-2-1), and BUN (#C013-2-1) were purchased from Nanjing Jiancheng Bioengineering Institute (Nanjing, China).

### 2.8 Cell lines and cell culture

The Nthy-ori 3–1 cell line (SV40-immortalized normal human thyroid epithelial cells) and the human thyroid carcinoma cell lines TPC-1 and B-CPAP were obtained from HyCyte^®^ (Shanghai, China). All cell lines were routinely tested for mycoplasma contamination prior to experimentation. Cells were maintained in DMEM supplemented with 10% fetal bovine serum (FBS), 100 U/mL penicillin, and 100 U/mL streptomycin (Cox9X@, China), and incubated at 37°C in a humidified atmosphere containing 5% CO_2_.

### 2.9 RNA interference, plasmid transfection, and drug treatment

TPC-1 and B-CPAP cells were seeded in 24 well at 2 x 10^5^ cell and transfected when they reached 60–70% confluence. For PLEC knockdown, cells were transfected with a final siRNA concentration of 50 nM using 50 μL Lipofectamine 2000 (Invitrogen, USA) per well. The PLEC-targeting sequences were siPLEC-1, 5′-UCAUCAUCGAGAUCAUUGA-3′; siPLEC-2, 5′-AGAGGUUUGCGAAACAGUA-3′; and siPLEC-3, 5′-AGCUCAAGAUCUCCUAUAA-3′. A scrambled siRNA without known complementarity to a human transcript was used as the negative control. The siRNA–Lipofectamine complexes were prepared in 50 μL of Opti-MEM I Reduced Serum Medium, incubated for 20 min at room temperature, and added to the cells for 6 h before replacement with complete culture medium.

For overexpression experiments, the PLEC expression plasmid construct contained no tag and was obtained from MIAOLING PLASMID (Wuhan, China). Cells were transfected with 0.8 μg plasmid and 2.0 μL Lipofectamine 2000 per well of a 24-well. The culture medium was replaced after 6 h. PLEC overexpression was assessed by RT-qPCR at 48 h after transfection and by Western blotting at 48 h.

Troglitazone was dissolved in DMSO and diluted in culture medium immediately before use. Unless otherwise specified, cells were exposed to 10 μM Troglitazone for 48 h, and the final DMSO concentration was identical in all groups. In rescue experiments, cells were transfected with the PLEC expression plasmid for 24 h before the addition of Troglitazone and were then exposed to Troglitazone for 48 h. The experimental groups were empty-vector plus vehicle (Control group), empty-vector plus Troglitazone (Troglitazone group), and PLEC-overexpression plus Troglitazone (Troglitazone + PLEC-OE group). Each experiment included three independent biological replicates, with three to five technical replicates per condition where applicable.

### 2.10 Functional assays

For the MTT assay, TPC-1 and B-CPAP cells were seeded in 96-well plates at 5,000 cells per well in 200 μL complete medium and allowed to attach for 24 h. Cells were then subjected to siRNA transfection, plasmid transfection, Troglitazone treatment, or the indicated combination treatment. Cell viability was measured at 0, 24, 48, and 72 hr. after transfection or after 48 h of Troglitazone exposure. MTT solution 5 mg/mL was added at 10 μL per well, and cells were incubated for 2 h at 37°C. The medium was removed, and the formazan crystals were dissolved in 150 μL DMSO. Absorbance at 570 nm was measured using a microplate reader with 570 nm as the reference. Blank wells containing medium and MTT reagent, but no cells were included. Cell viability was calculated as follows: viability (%) = (ODtreatment − ODblank)/(ODcontrol − ODblank) × 100. Three independent biological experiments were performed, with three to five technical wells per condition in each experiment.

For colony-formation assays, cells were harvested at 48 h after transfection or treatment and reseeded in 12-well at 1,500 cells per well. Cells were cultured for 10–14 days, with the medium replaced every 5 days. Colonies containing more than 50 cells were fixed with 4% paraformaldehyde for 30 min, stained with 0.5% crystal violet for 15 min, imaged, and counted using ImageJ software. Three independent biological experiments were performed.

For EdU incorporation assays, cells were seeded in 24-well plates at 2 × 10^4^ cells per well and subjected to the indicated transfection or treatment. At 48 h, EdU working solution from the EdU staining kit was added, and cells were incubated for 2 h at 37°C. Cells were fixed with 4% paraformaldehyde for 15 min, permeabilized with 0.3% Triton X-100 in PBS for 15 min, and processed according to the manufacturer’s instructions. Images were acquired using a Phenix fluorescence microscope (Shangrao, China). The percentage of EdU-positive cells was calculated from five randomly selected non-overlapping fields per sample. Three independent biological experiments were performed.

For Transwell migration assays, 5 × 10^5^ treated cells were suspended in 200 μL serum-free medium and added to the upper chamber of Transwell inserts. The lower chamber contained 600 μL complete medium supplemented with 10% FBS as a chemoattractant. No Matrigel coating was used. After 24 h, non-migrated cells were removed from the upper surface of the membrane. Cells that had migrated to the lower surface were fixed with 4% paraformaldehyde for 30 min and stained with 0.1% crystal violet for 30 min. Migrated cells were counted in five randomly selected fields per insert using microscope. Three independent biological experiments were performed.

For acridine-orange/ethidium-bromide staining, cells were seeded in 2 × 10^4^ at 24-well. Cells were incubated with an acridine-orange/ethidium-bromide mixture at 10μL/mL for 2–3 mins at room temperature in the dark. After washing with PBS, fluorescence images were acquired using a Phenix fluorescence microscope. Apoptotic cells were quantified in five randomly selected non-overlapping fields per sample by an investigator. Three independent biological experiments were performed.

For qRT-PCR, total RNA was extracted for cell culture and tissue samples using TRIzol reagent. To conduct reverse-transcribed by primescript RT reagent kits followed by Applied Biosystems 7500 Fast Real-Time PCR System and quantified using SYBR Green qPCR Mix. Relative mRNA expression was calculated using the 2^-ΔΔ^Ct method, with GAPDH as the internal reference. qRT-PCR was performed using 3 independent biological replicates, with 3–5 technical replicates per sample. Primer sequences for qRT-PCR were as follows:

PLEC (Hum):
forward, 5′-ATCTCCCTCTTCCAGGCCAT-3′,
reverse, 5′-CTCCTCAGGGTCGATGATGC-3′.
GAPDH (Hum):
forward, 5′-GTCAAGGCTGAGAACGGGAA-3′,
reverse, 5′-AAATGAGCCCCAGCCTTCTC-3′.

Primer oligonucleotides were synthesized by Servicebio (Wuhan, China). RT-qPCR reporting was structured according to the MIQE 2.0 recommendations.

For Western blotting, total protein was extracted using RIPA buffer supplemented with protease inhibitors and quantified using a BCA assay. A total of 20-40 μg protein per lane was separated by 3% SDS–PAGE and transferred to nitrocellulose membranes. Membranes were blocked with 5% non-fat milk for over-night and then incubated with anti-PLEC (1:500) antibodies at 4 °C for two days. After washing, membranes were incubated with HRP-conjugated secondary antibody for 1 h. Signals were visualized using West ECL substrate, and band intensities were quantified using ImageJ. Three independent biological experiments were performed.

### 2.11 Candidate-compound screening and molecular docking

Candidate compounds associated with PLEC were retrieved from DGIdb (https://dgidb.org/, accessed on Nov/1/2025) and DrugMap (https://drugmap.idrblab.net/, accessed on Nov/1/2025) using PLEC as the query gene. Drug–gene interaction records were retained when approved-drug status. Duplicate compounds and compounds without an available three-dimensional structure were removed. The complete screening and filtering workflow is provided in **Table 1**.

**Table 1:** Binding energy of six active components and PLEC.

Target	Candidate compound	Structure	Molecular weight (g/mol)	Predicted binding energy (kcal/mol)
PLEC (1MB8)	Troglitazone	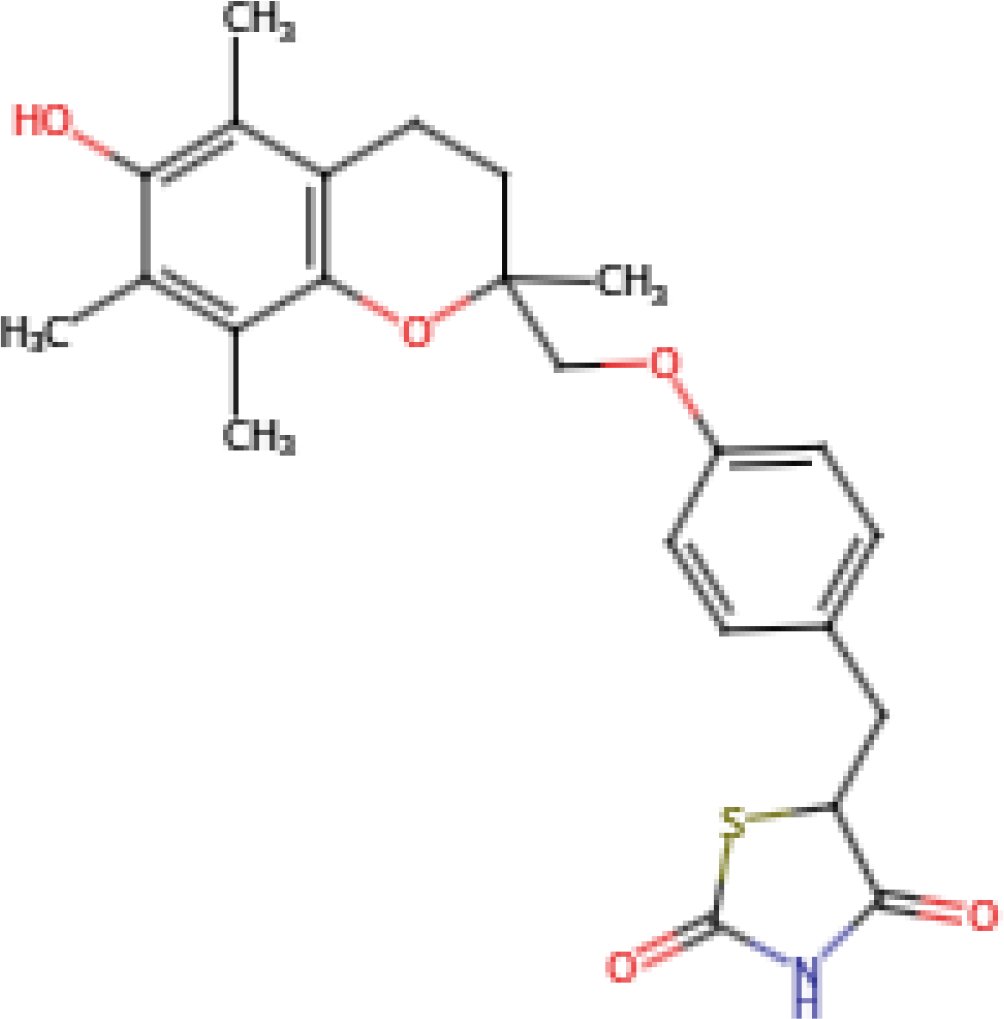	441.5	-7.5
PLEC (1MB8)	Fulvestrant	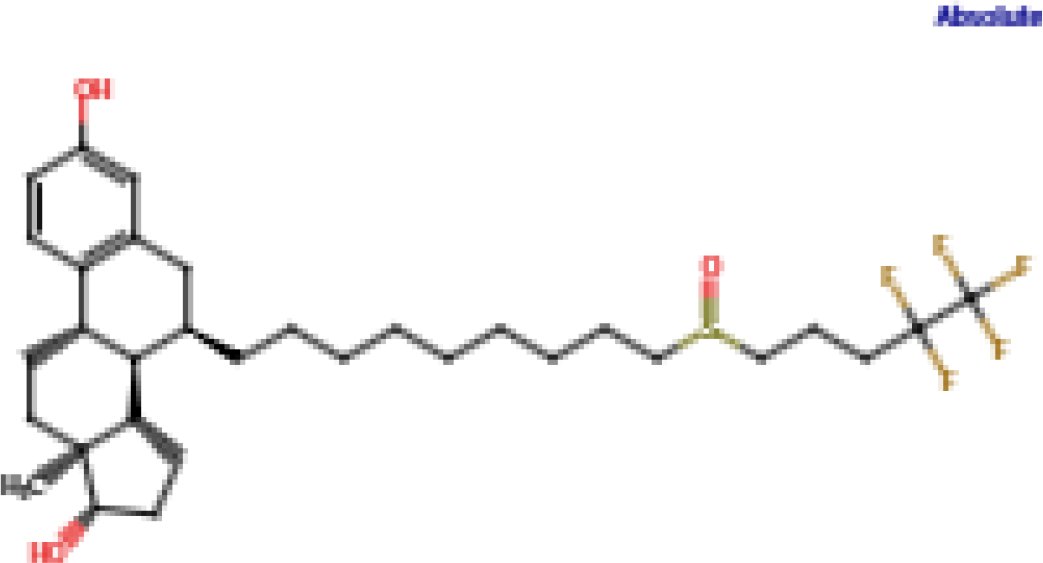	606.8	-6.6
PLEC (1MB8)	Niclosamide	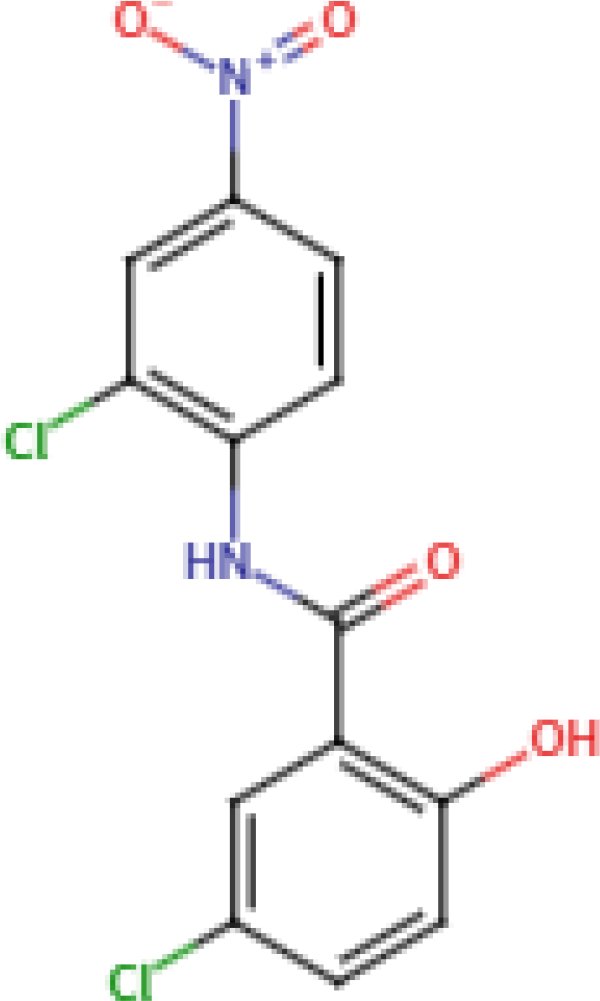	327.12	-6.4
PLEC (1MB8)	Cidofovir	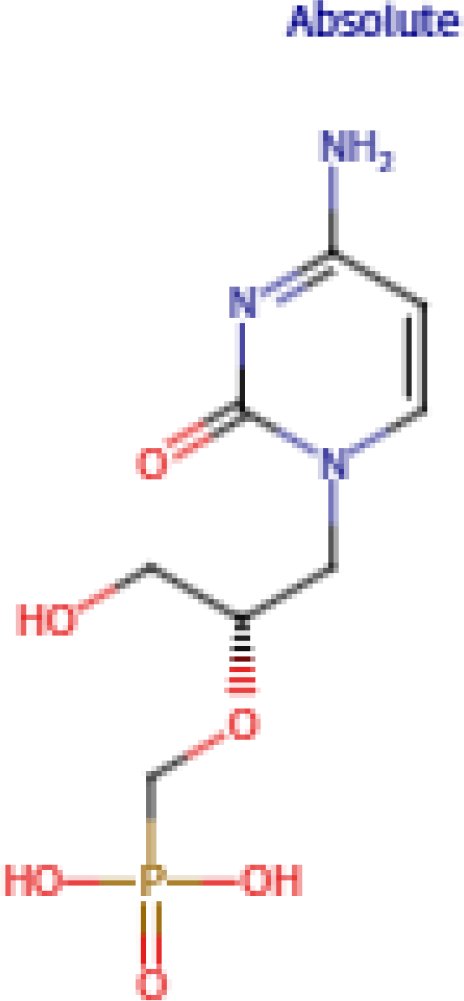	279.19	-5.3
PLEC (1MB8)	Zoledronate	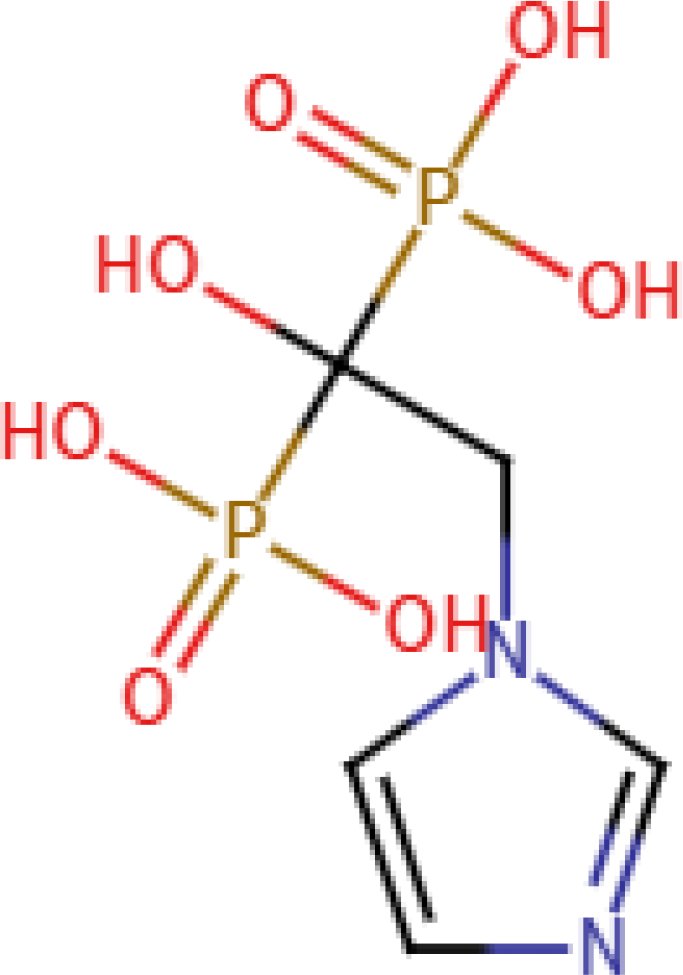	272.09	-5.1
PLEC (1MB8)	Clodronate	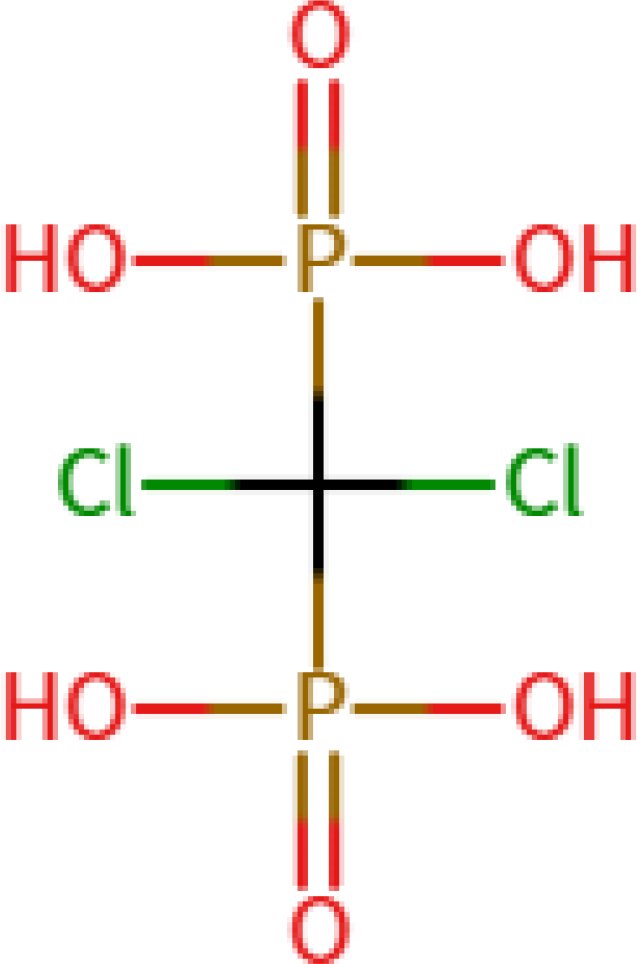	244.89	-4.6

To validate ligand-target interactions, previously identified bioactive compounds were docked against the PLEC protein using AutoDock Vina. Protein structures were sourced from the RCSB PDB (http://www.rcsb.org/) with crystallographic resolution ≤ 2.5 Å. Co-crystallized ligands and water molecules were removed from each protein structure using PyMOL 2.5.8. A grid box encompassing the entire protein surface and putative binding sites was generated. Molecular interactions were visualized and analyzed using PyMOL 2.5.8 and Discovery Studio 2024. Binding energies ≤ -5.0 kcal/mol indicated strong binding affinity.

### 2.12 Molecular dynamics simulation

MD simulations were performed using Amber 22 to characterize the binding mode and energy. These simulations targeted the ligand-receptor complex exhibiting the lowest binding energy, as identified in prior molecular docking. The simulation system was solvated in a TIP3P water box and neutralized with counter-ions. Following energy minimization, gradual heating to 300 K occurred over a period of 500 ps. Subsequent equilibration proceeded at 300 K within the canonical (NVT) ensemble. Finally, production MD simulations lasting 100 ns were conducted under the isothermal-isobaric (NPT) ensemble. Bond constraints for hydrogen atoms were enforced using the SHAKE algorithm. Trajectory data analysis utilized AmberTools.

### 2.13 Cellular thermal shift assay

B-CPAP cells were harvested, washed with ice-cold PBS, and resuspended in ice-cold PBS supplemented with 1× protease inhibitor cocktail. Cells were lysed by three freeze–thaw cycles, and the lysates were clarified by centrifugation at 20,000 × g for 20 min at 4°C. The soluble lysates were collected, adjusted to equal protein concentrations, and divided equally into DMSO- and Troglitazone (10 μM, with an equivalent final concentration of DMSO)-treated groups. Lysates were incubated room temperature for 1 h. After incubation, aliquots were heated at 40, 43, 46, 52, 55, 58, and 61°C for 3 min, followed by immediate cooling on ice. Heat-induced insoluble protein aggregates were removed by centrifugation at 20,000 × g for 20 min at 4°C. Equal volumes of the soluble supernatants were mixed with SDS loading buffer and separated by low-percentage SDS-PAGE followed by western blotting. PLEC protein levels were detected using an anti-PLEC antibody (1:1,000). Band intensities were quantified using ImageJ and normalized to the signal at 40°C. Experiments were performed with three independent biological replicates. Melting curves were generated from normalized band intensities, and differences between DMSO- and Troglitazone-treated groups were analyzed by two-way ANOVA followed by Sidak’s multiple-comparison test. *P* < 0.05 was considered statistically significant.

### 2.14 *In vivo* tumorigenicity assay in nude mice

All animal experiments were performed in accordance with the ARRIVE guidelines and were approved by the Animal Ethics Committee of the Second Affiliated Hospital of Harbin Medical University (Approval No. YJSDW2024-272). BALB/c nude mice (male, 4–6 weeks old) were purchased from Liaoning Changsheng Laboratory Animal Technology Co., Ltd. (Shenyang, China). Subcutaneous xenografts were established by injecting 2 x 10^7^ B-CPAP cells into the right dorsal flank.

After tumor establishment, ten mice were assigned unique identification numbers and randomly allocated in a 1:1 ratio to the two groups using a computer-generated random-number sequence: vehicle group (5% DMSO, n = 5) and Troglitazone-treated group (150 mg/kg Troglitazone in 5% DMSO, n = 5). Compounds were administered by intraperitoneal injection every 3 days for a total of three doses. Tumor length and width were measured every 3 days using calipers, and tumor volume was calculated as volume = length × width^2^/2.

Humane endpoints included tumor volume approaching approximately 2 × 10^3^ mm^3^, severe ulceration, rapid body-weight loss, impaired mobility, or other signs of distress according to institutional animal welfare criteria. Mice were euthanized by CO_2_ asphyxiation after the final administration or earlier if humane endpoints were reached. Tumors were harvested for weighing, measurement, and histological evaluation. Blood was collected for serum biochemical analysis of ALT, AST, CRE, and BUN. Major organs, including the liver, lung, kidney, and heart, were collected for H&E staining. For tumor volume and tumor weight analyses, n refers to the number of mice per group. For biochemical and histological analyses, n refers to 5 for one mouse.

### 2.15 Statistical analysis

Statistical analyses were performed using R v4.3.1 and GraphPad Prism 9.0, as appropriate. Data are presented as the mean ± SD unless otherwise stated. For *in vitro* experiments, n represents the number of independent biological replicates, and technical replicates were used only for measurement precision. For animal experiments, n represents the number of mice per group unless otherwise specified.

Normality was assessed using the Shapiro-Wilk test. For normally distributed data, comparisons between two groups were performed using Student’s t-test, whereas comparisons among more than two groups were performed using one-way ANOVA followed by Tukey’s *post-hoc* test. For non-normally distributed data, the Mann-Whitney U test or Kruskal-Wallis test followed by Dunn’s *post-hoc* test was used as appropriate. For repeated tumor-volume measurements, two-way repeated-measures ANOVA followed by Sidak’s multiple-comparison test was used.

For bioinformatics analyses involving multiple comparisons, adjusted *P* values were calculated using the Benjamini-Hochberg false discovery rate method unless otherwise indicated. For GO, GSEA, immune infiltration, and subtype-associated analyses, adjusted P values or false discovery rate values are reported where applicable. ROC analyses were performed using the pROC package, and AUC values with 95% confidence intervals were reported. A two-sided *P* value < 0.05 was considered statistically significant.

The previous statement that survival analysis was performed has been removed because survival results were not presented in the current manuscript.

For bioinformatics analyses involving multiple gene, pathway, or immune-cell comparisons, *P* values were adjusted using the Benjamini-Hochberg false discovery rate method unless otherwise specified. The R packages used in the bioinformatics analyses included ConsensusClusterPlus (v1.64.0), clusterProfiler (v4.6.2), maftools (v2.16.0), GSEA (v1.48.2) and pROC (v1.18.4). GISTIC 2.0 was used for copy number variation analysis.

## 3 Results

### 3.1 Mutational landscape of thyroid cancer molecular subtypes

**Figure 3:**
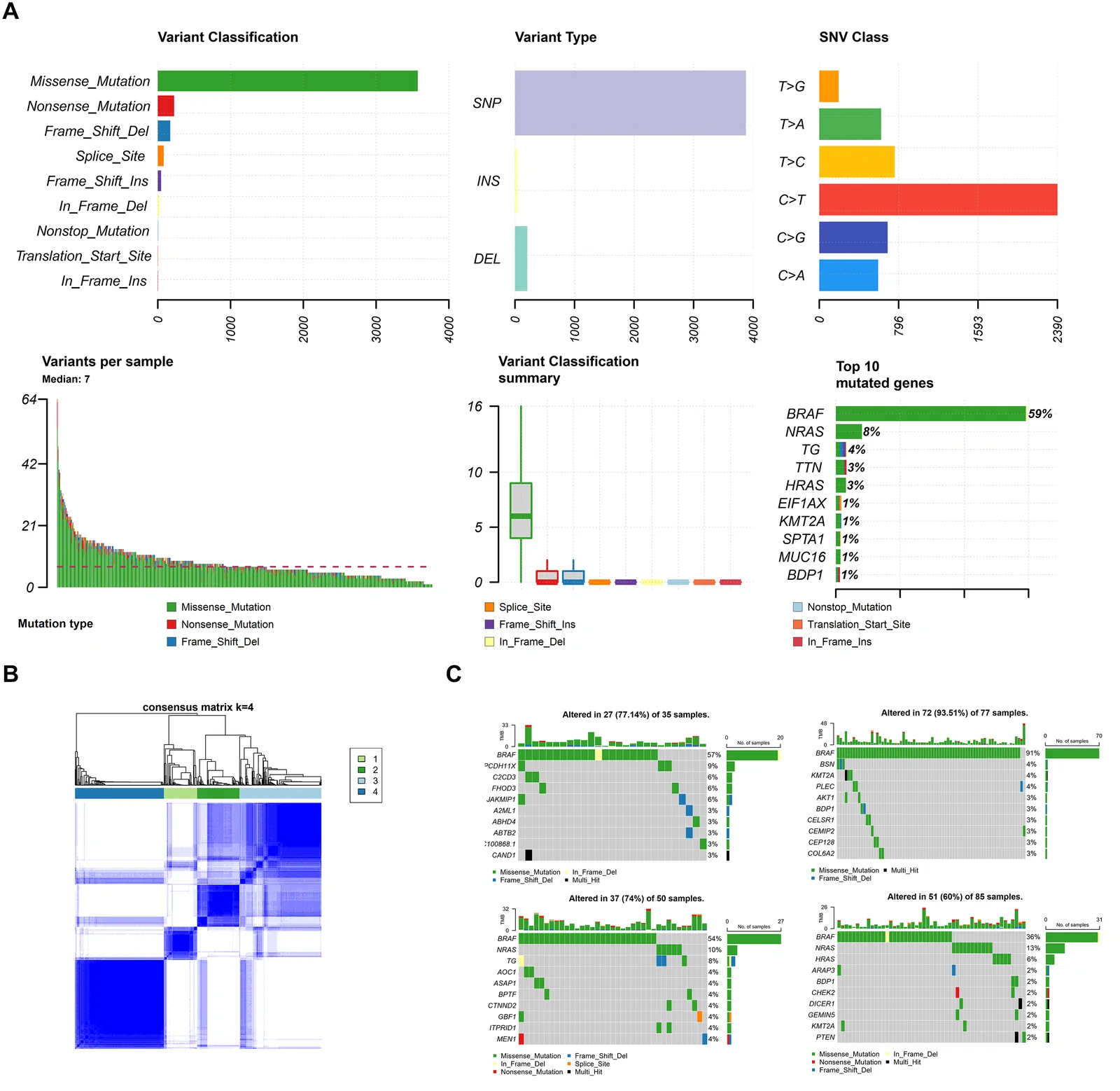
Molecular subtyping and mutational landscape of PTC. **(A)** Mutational profile of thyroid cancer samples from TCGA, including mutation type distribution, variant classification, single-nucleotide variant class, per-sample mutation burden, tumor mutation burden, and the top mutated genes. **(B)** Consensus clustering heatmap based on transcriptomic profiles, defining four molecular subtypes (A1-A4). **(C)** Subtype-specific mutation profiles for A1-A4. Color-coded tiles indicate mutation types. Gene symbols are shown on the left, and mutation frequencies are shown on the right.

To characterize transcriptomic heterogeneity in PTC, unsupervised consensus clustering was applied to the gene-expression profiles of 512 eligible TCGA-THCA primary PTC samples (**Figure 3A**). The clustering input consisted of all expressed genes after normalization. Importantly, the A1–A4 groups were defined exclusively from transcriptomic data and were not generated using somatic mutation or copy-number variation (CNV) profiles. Consensus clustering was performed using ConsensusClusterPlus with unsupervised clustering resampling iterations. Evaluation of the consensus matrices together with cumulative distribution function supported (k=4) as an appropriate solution, resulting in subtypes A1–A4 containing, respectively (**Figure 3B**).

The clustering analysis was restricted to samples meeting the histological criteria of PTC. Clinical variables were not used to define the clusters but were examined subsequently in relation to the transcriptome-derived subtype assignments. Because the present study did not perform a formal concordance analysis between A1–A4 and established BRAF-like, RAS-like, or PDTC-like classifications, the four groups were retained as study-specific transcriptomic clusters and should not be considered equivalent to these previously defined molecular categories.

After transcriptomic subtype assignment, somatic mutation profiles were examined independently using GDC masked somatic mutation annotation format files from 4 subtype-matched PTC samples. Germline variants were not included in the masked somatic mutation dataset. Variants were processed using maftools. The mutation landscape was summarized according to variant classification, variant type, single-nucleotide variant class, per-sample mutation burden, and the frequencies of recurrently mutated genes. Missense mutations represented the predominant variant classification, with an average of seven mutations per sample, and single-nucleotide polymorphisms were more frequently observed than insertions or deletions. *BRAF* was the most frequently mutated gene (59%), followed by *NRAS* (8%), *TG* (4%), *TTN* (3%), and *HRAS* (3%).

Descriptive comparison of the subtype-specific mutation profiles showed that *BRAF* remained the most frequently mutated gene across A1–A4. Within the displayed mutation profiles, *CAND1* frameshift deletions and *PCDH11X* missense variants were observed in A1, whereas *PTEN* alterations were observed among A4 samples (**Figure 3C**). Because formal gene-level comparisons of mutation frequencies among the four subtypes were not performed, these findings are reported as observed mutation patterns rather than statistically significant mutation enrichment.

### 3.2 Functional and Microenvironmental Characteristics

Subtype-specific CNV gains included 2p16.1/19p13.3 (A1), 20q13.2 (A2), 8p22 (A3), and 4q31.21 (A4) (**Figure 1A**). GO enrichment indicated subtype A1 alterations were enriched in sprouting angiogenesis pathways, while A3 showed STAT pathway enrichment. GSEA revealed significant upregulation of immune-related pathways in subtype A3 (**Figure 1B**), potentially linked to STAT-associated mutations. CIBERSORT analysis demonstrated statistically distinct immune infiltration patterns between A3/A4 subtypes (*P* < 0.05 by Kruskal-Wallis test; **Figure 1C**). In GEO validation datasets, A3-specific mutation markers demonstrated superior tumor-discriminatory capacity (AUC = 0.92, 95%CI:0.85-0.97; **Figure 1D**), supporting the clinical relevance of apoptosis-related subtypes. To further connect the molecular subtype analysis with subsequent candidate-gene prioritization, we examined PLEC expression across the A1-A4 subtypes. PLEC expression was compared among the four molecular subtypes using wilcoxon rank-sum test. PLEC expression was significantly different across A1-A4 subtypes, with relatively higher expression observed in A3 compared with A1, A2 and A4 (*P*<0.0001; **Figure 1E**). We also assessed the relationship between PLEC expression and subtype-related clinicopathological features. PLEC expression was associated with Pathologic N stage(*P* = 0.009), Neoplasm location(*P* = 0.011), Histological type (*P*=< 0.001) Residual tumor(*P* = 0.003), supporting its further evaluation as a candidate molecule linked to aggressive PTC phenotypes (**Table 2**).

**Table 2:** Relationship between PLEC expression and clinical characteristics in 512 PTC patients from the TCGA database.

Characteristics	Low expression of PLEC	High expression of PLEC	*P* value
n	256	256	
Gender, n (%)			0.371
Female	182 (35.5%)	191 (37.3%)	
Male	74 (14.5%)	65 (12.7%)	
Age, n (%)			0.250
<= 45	115 (22.5%)	128 (25%)	
> 45	141 (27.5%)	128 (25%)	
Pathologic N stage, n (%)			0.009
N0	128 (27.7%)	101 (21.9%)	
N1	102 (22.1%)	131 (28.4%)	
Neoplasm location, n (%)			0.011
Bilateral	36 (7.1%)	52 (10.3%)	
Isthmus	6 (1.2%)	16 (3.2%)	
Left lobe	89 (17.6%)	89 (17.6%)	
Right lobe	123 (24.3%)	95 (18.8%)	
Histological type, n (%)			< 0.001
Classical	159 (31.1%)	207 (40.4%)	
Follicular	72 (14.1%)	29 (5.7%)	
Tall Cell	19 (3.7%)	17 (3.3%)	
Other	6 (1.2%)	3 (0.6%)	
Residual tumor, n (%)			0.003
R0	203 (45.1%)	189 (42%)	
R1&R2	18 (4%)	40 (8.9%)	

These findings provided a rationale for prioritizing PLEC for downstream validation. Importantly, the molecular subtype analysis was used as an exploratory framework to understand PTC heterogeneity and guide candidate selection, rather than as direct evidence of subtype-specific drug response.

### 3.3 PLEC is up-regulated in PTC and is associated with unfavorable clinicopathological features

Based on the subtype-informed candidate prioritization described above, we focused on PLEC for further validation. PLEC was selected because it was upregulated in PTC, showed association with A3, and was associated with unfavorable clinicopathological features. Using RNA-seq data from TCGA-THCA and independent GEO datasets, we observed increased PLEC expression in PTC samples compared with non-tumor thyroid tissues (**Figures 2B, C**). Consistently, GSE33630 and GSE60542 also showed elevated PLEC levels in tumor tissues (**Figures 2D, E**). ROC analysis suggested preliminary diagnostic relevance of PLEC expression for distinguishing PTC tissues from non-tumor thyroid tissues (**Figure 2F**). However, these findings should be interpreted as exploratory and require further validation in larger independent cohorts. We next performed preliminary validation in our clinical cohort by quantifying PLEC mRNA in 15 paired PTC and adjacent non-tumorous tissues (**Figure 2G**). Immunohistochemical staining and semi-quantitative scoring of the same 15 paired samples further supported higher PLEC protein expression in PTC tissues than in matched adjacent tissues (**Figures 2H, I**).

### 3.4 PLEC knockdown suppresses PTC cell proliferation and migration and induces apoptosis

**Figure 4:**
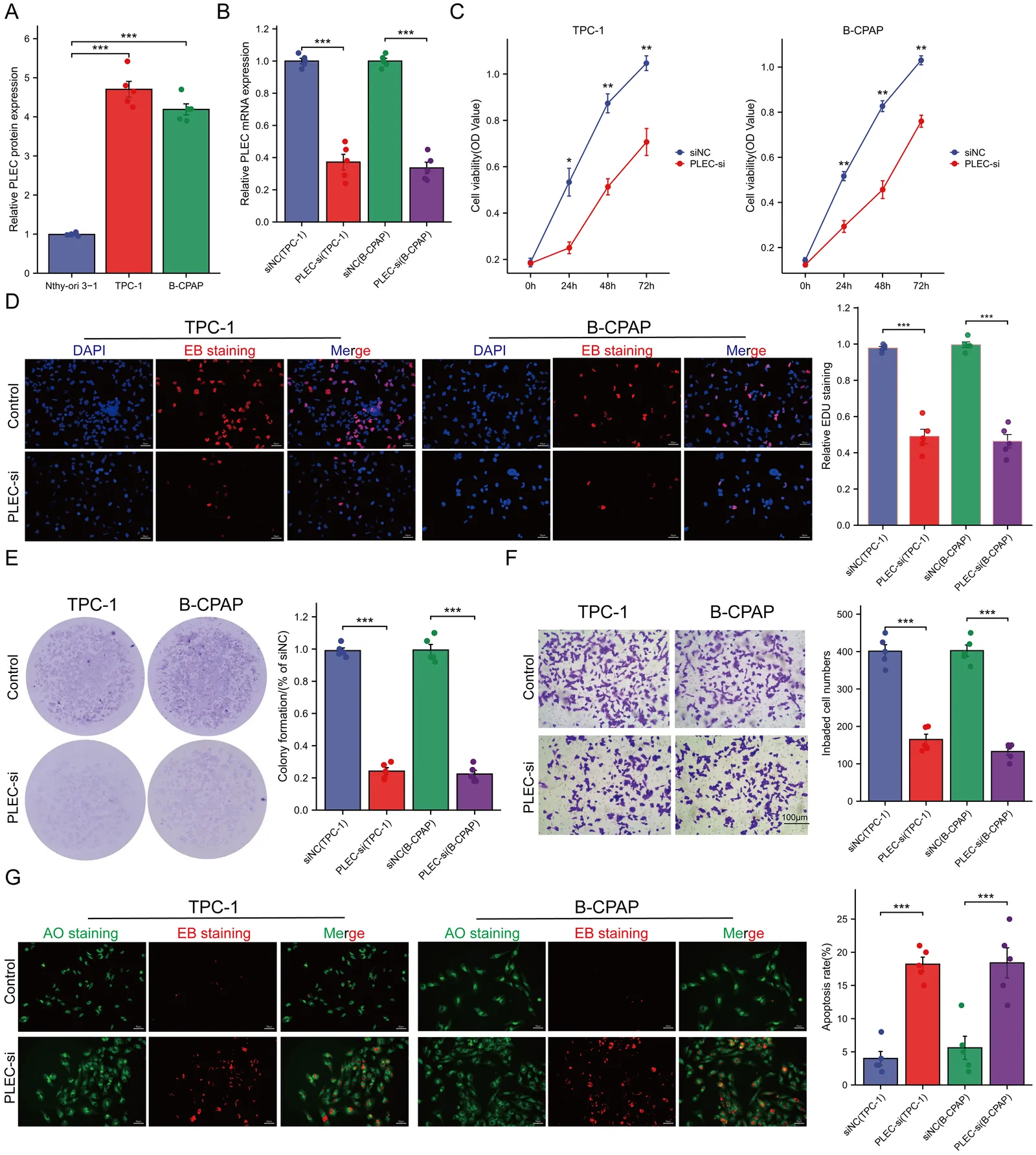
PLEC knockdown suppresses PTC cell proliferation and migration and induces apoptosis. **(A)** PLEC expression in Nthy-ori 3-1, TPC-1, and B-CPAP cells was analyzed by RT-qPCR. **(B)** PLEC knockdown efficiency in TPC-1 and B-CPAP cells was analyzed by RT-qPCR after transfection with PLEC siRNA. **(C)** MTT assay showing cell viability after PLEC knockdown. **(D, E)** EdU staining and colony formation assays showing proliferative capacity after PLEC knockdown. **(F)** Transwell migration assay showing invasive ability after PLEC knockdown. **(G)** AO/EB staining showing apoptosis after PLEC knockdown. Data are presented as the mean ± SD from 3 independent biological replicates. Statistical significance was assessed using Student’s t-test. *P < 0.05; **P < 0.01; ***P < 0.001 compared with siNC. Student’s t-test, whereas comparisons among more than two groups were performed using one-way ANOVA followed by Tukey’s *post-hoc* test.

To investigate the functional contribution of PLEC to PTC, Nthy-ori 3–1 cells were used as a normal thyroid epithelial control, whereas TPC-1 and B-CPAP cells served as human PTC models. RT–qPCR showed that PLEC expression was markedly higher in PTC cell lines than in Nthy-ori 3–1 cells, and siRNA-mediated silencing efficiently reduced PLEC levels (**Figures 4A, B**). To determine whether PLEC regulates malignant phenotypes, TPC-1 and B-CPAP cells were transfected with negative-control siRNA (siNC) or PLEC-targeting siRNA. PLEC depletion significantly suppressed cell growth (**Figure 4C**), and this inhibitory effect was corroborated by EdU incorporation and colony formation assays (**Figures 4D, E**). In addition, Transwell migration assays demonstrated a pronounced reduction in invasive capacity following PLEC knockdown compared with siNC-treated cells (**Figure 4F**). Apoptosis was assessed using AO/EB staining, which revealed that PLEC silencing increased apoptotic cell death in both PTC cell lines (**Figure 4 G**). Together, these results suggest that PLEC promotes PTC cell proliferation and migration while restraining apoptosis.

### 3.5 Screening of candidate compounds potentially associated with PLEC in PTC

**Figure 5:**
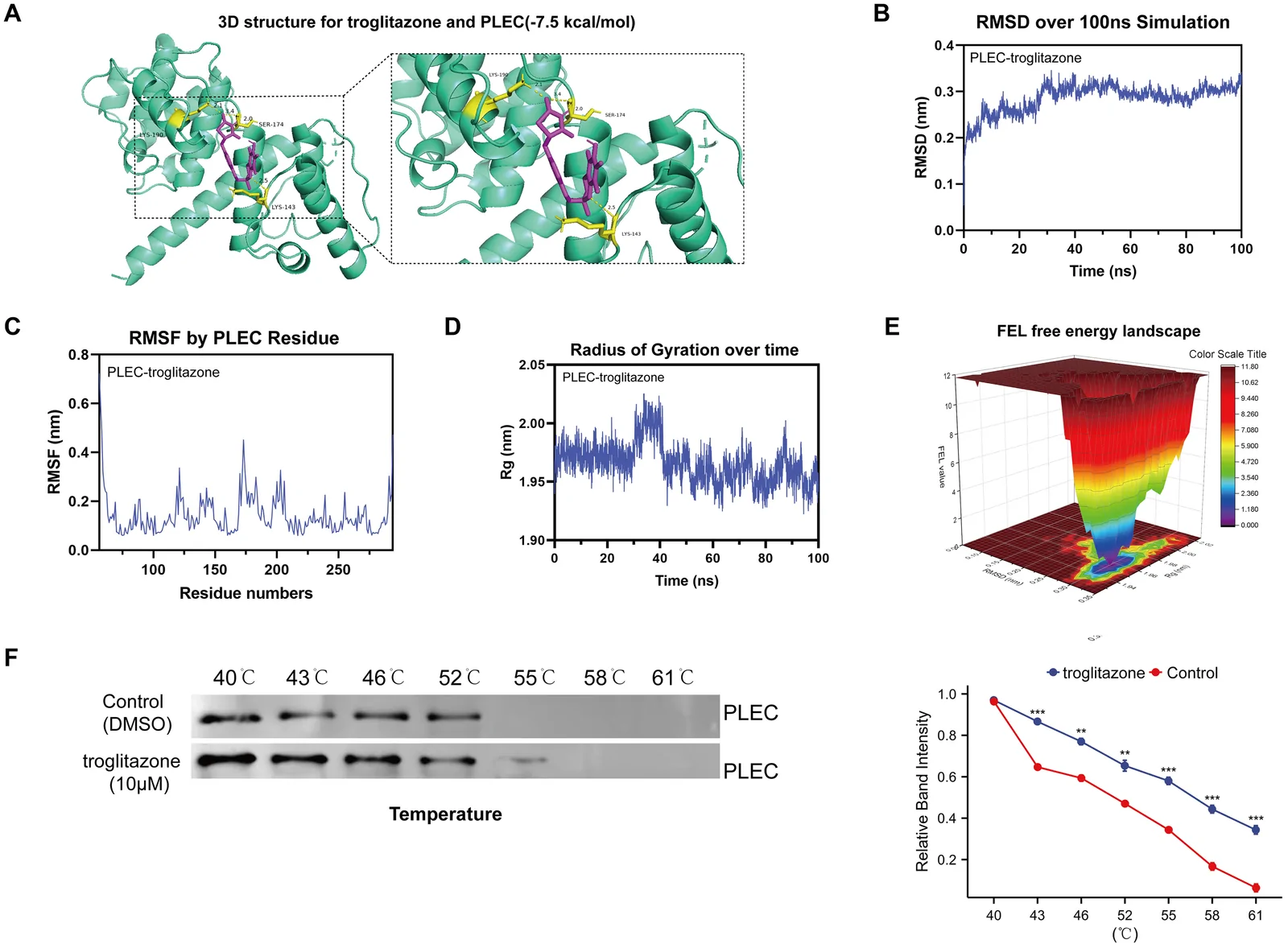
Computational prediction and CETSA-based assessment of the putative Troglitazone-PLEC interaction. **(A)** Three-dimensional and two-dimensional interaction diagrams of the Troglitazone-PLEC complex. Binding energy is shown in kcal/mol. **(B)** RMSD analysis of the Troglitazone-PLEC complex during molecular dynamics simulation. **(C)** RMSF analysis showing residue-level flexibility. **(D)** Radius of gyration analysis showing structural compactness. **(E)** FEL free energy landscape analysis of the Troglitazone-PLEC complex. **(F)** CETSA analysis of PLEC protein thermal stability in DMSO- and Troglitazone-treated samples under the indicated temperature conditions. RMSD, root mean square deviation; RMSF, root mean square fluctuation; Rg, radius of gyration; CETSA, cellular thermal shift assay.

Potential candidate compounds associated with PLEC were first identified by querying drug-gene interaction resources, including DGIdb and DrugMap. Six FDA-approved or previously characterized compounds were selected for further computational assessment: cidofovir, clodronate, fulvestrant, niclosamide, Troglitazone, and zoledronate. Prior work has implicated several drug candidates in regulating apoptosis and tumor-related phenotypes across thyroid and breast cancer models. In our data, molecular docking was then performed to estimate the predicted binding energy between these compounds and PLEC. Among the screened compounds, Troglitazone showed the lowest predicted binding energy with PLEC (-7.5 kcal/mol), suggesting that it may have a putative interaction with PLEC (**Table 1**; **Figure 5A**).

To further explore the stability of the predicted Troglitazone-PLEC complex, molecular dynamics simulation was performed. The RMSD, RMSF, and radius of gyration analyses suggested that the predicted complex remained relatively stable during the simulation period under the modeled conditions (**Figures 5B–E**). These computational analyses should be interpreted as hypothesis-generating evidence rather than definitive proof of direct biochemical binding.

We next performed CETSA to determine whether Troglitazone treatment altered the thermal stability of PLEC. Compared with the DMSO-treated control, Troglitazone treatment (10 μM) increased the detectable soluble PLEC signal at higher temperatures under the tested conditions (**Figure 5F**). Quantitative analysis showed increased relative PLEC band intensity at 55°C (*P*<0.05). These findings support a putative interaction between Troglitazone and PLEC and suggest that Troglitazone treatment may increase PLEC thermal stability under the tested conditions. However, additional orthogonal biochemical assays are required to confirm direct target engagement.

### 3.6 Troglitazone shows anti-PTC effects associated with PLEC modulation

**Figure 6:**
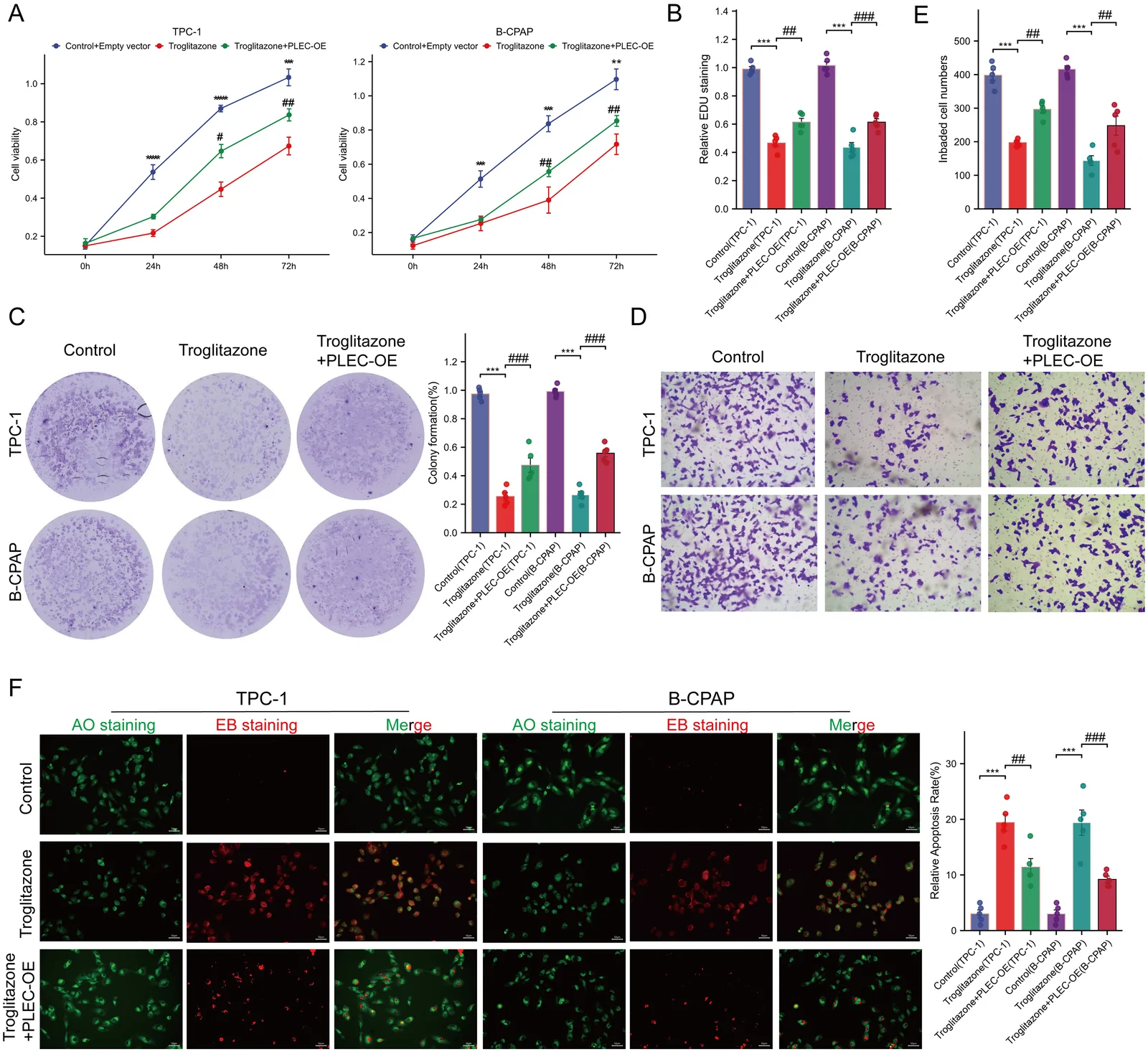
Troglitazone suppresses PTC malignant phenotypes in a PLEC-associated manner. **(A)** MTT assay showing cell viability after treatment with vehicle control, Troglitazone, or Troglitazone plus PLEC overexpression. **(B, C)** EdU staining and colony formation assays showing cell proliferation under the indicated conditions. **(D, E)** Transwell assay showing cell migration under the indicated conditions. **(F)** AO/EB staining showing apoptosis under the indicated conditions. Troglitazone was used at 10 μM for 48 h based on preliminary IC_50_ result. Data are presented as the mean ± SD from 3 independent biological replicates. Statistical analysis was performed using one-way ANOVA followed by Tukey’s *post-hoc* test. **P < 0.01; ***P < 0.001 versus control group; ^##^*P* < 0.01; ^###^*P* < 0.001 versus Troglitazone group.

To evaluate the anti-PTC effect of Troglitazone *in vitro*, TPC-1 and B-CPAP cells were treated with Troglitazone at 10 μM for 48 h, based on preliminary IC_50_ results. As shown in **Supplementary Figure 2**, Troglitazone treatment reduced cell viability, inhibited colony formation and migration, and increased apoptotic cell death under the tested conditions. To explore the anti-PTC effect of Troglitazone. We first test the anti-tumor effect of Troglitazone for PTC (**Supplementary Figure 2**). Our data showed that Troglitazone (10 μM for 48 hrs.) reduced PTC cell viability and migration and increased apoptosis. Next, we explored the relationship between Troglitazone and PLEC in PTC cells. We employed EdU staining, colony formation, MTT, AO/EB staining as well as Transwell assay to test the anti-PTC effect of PLEC/Troglitazone, we treat TPC-1 and B-CPAP cells with Empty vector, Troglitazone or Troglitazone plus PLEC-overexpression. As shown in **Figure 6A**, MTT was employed to test cell growth. PLEC overexpression partially attenuated the inhibitory effect of Troglitazone on cell growth. EdU staining and colony formation assays revealed that PLEC overexpression partially rescued the anti-proliferative effect of Troglitazone in PTC cells (**Figures 6B, C**). Transwell assay showed that PLEC overexpression partially restored invasive capacity in Troglitazone-treated PTC cells (**Figures 6 D, E**). AO/EB staining data shown PLEC-overexpression significantly rescues the apoptosis rate induced by Troglitazone in PTC cells (**Figure 6F**). Collectively, our findings indicate that PLEC may participate in the anti-PTC response associated with Troglitazone treatment.

### 3.7 Troglitazone suppresses xenograft growth without overt toxicity under the tested conditions

**Figure 7:**
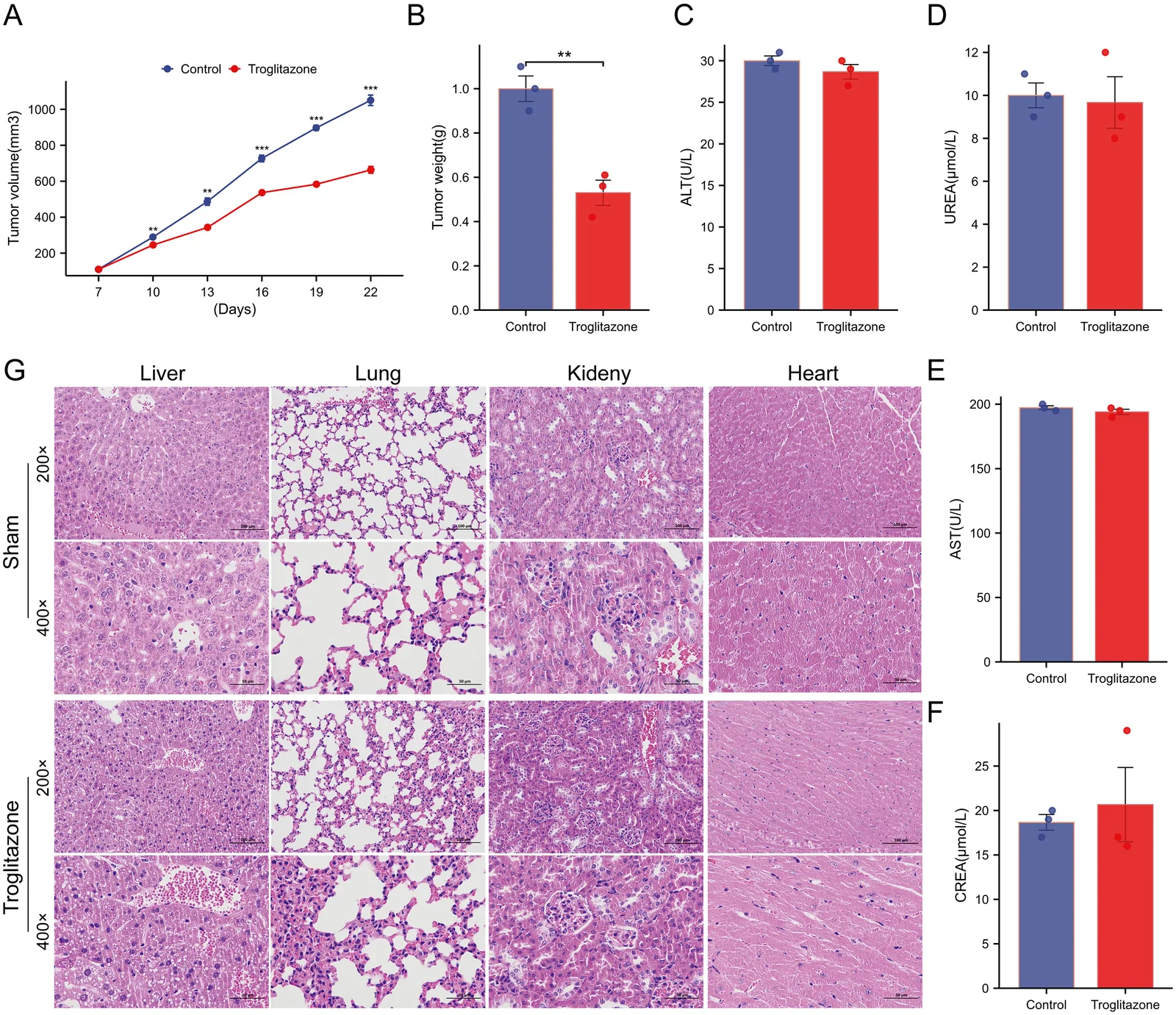
Troglitazone suppresses xenograft tumor growth without overt toxicity under the tested conditions. **(A, B)** Tumor growth curves and tumor weights in nude mice bearing B-CPAP xenografts treated with vehicle or Troglitazone. **(C-F)** Serum ALT, AST, CRE, and BUN levels in vehicle- and Troglitazone-treated mice. **(G)** Representative H&E staining of liver, lung, kidney, and heart tissues from vehicle- and Troglitazone-treated mice. Scale bar = 100 μm. For tumor volume and tumor weight analyses, n = 5 mice per group. For serum biochemical and histological analyses, n =5 per group. Data are presented as the mean ± SD. Statistical analysis was performed using Student’s t-test. **P < 0.01 compared with the vehicle group. ALT, alanine aminotransferase; AST, aspartate aminotransferase; CRE, creatinine; BUN, blood urea nitrogen.

To evaluate the *in vivo* relevance of Troglitazone treatment, we established a subcutaneous B-CPAP xenograft mouse model. Based on the *in vitro* findings presented in **Supplementary Figure 2** and **Figure 6**, we further assessed whether Troglitazone could suppress xenograft tumor growth under the tested experimental conditions. Compared with the control group, Troglitazone treatment at 150 mg/kg significantly restrained tumor growth, with corresponding reductions in tumor volume and tumor weight (**Figures 7A, B**). No significant differences in body weight were observed between Troglitazone- and vehicle-treated mice, indicating good tolerability. In addition, plasma biochemical indices-including ALT, AST, CRE, and BUN-were comparable between groups, suggesting no over-hepatic or renal impairment following Troglitazone administration (**Figures 7C–F**). Consistent with these findings, the relative organ weights (heart, liver, lung, and kidney) did not differ significantly between groups, and H&E staining revealed no apparent histopathological abnormalities in these organs (**Figure 7G**). Collectively, these data indicate that Troglitazone inhibits B-CPAP xenograft tumor growth *in vivo* without detectable systemic toxicity under the conditions tested.

## 4 Discussion

This study establishes a comprehensive molecular taxonomy of thyroid carcinoma through integrated multi-omics analysis and identifies PLEC as a therapeutically actionable oncoprotein. The molecular subtype analysis provided an exploratory framework for understanding PTC heterogeneity and supported candidate-gene prioritization, rather than directly establishing subtype-specific therapeutic vulnerability. Our findings reveal that PLEC-associated malignant phenotypes may be attenuated by Troglitazone under the tested experimental conditions, an FDA-approved drug repurposed through rigorous computational and functional validation.

Consensus clustering delineated four molecularly distinct subtypes (A1-A4) with characteristic driver alterations: A1 tumors harbored co-occurring *CAND1 (*23), frameshift deletions and *PCDH11X (*24) missense mutations alongside canonical *BRAF* mutations (25), while A4 exhibited frequent *PTEN* inactivation – a finding with direct therapeutic implications given ongoing PI3K inhibitor trials. Crucially, subtype-specific CNV gains (e.g., 20q13.2 in A2) aligned with established oncogene loci (*BCL2L1*, *ZNF217*), explaining differential pathway activation patterns. The A3 subtype’s STAT pathway enrichment and immune hyperactivation (CIBERSORT; *P* < 0.05) provide mechanistic rationale for immunotherapy responsiveness, consistent with recent reports linking STAT signatures to PD-L1 expression in thyroid cancer (26, 27). Our validation of A4-specific markers’ diagnostic power (AUC = 0.92) surpasses existing molecular classifiers, enabling non-invasive stratification of apoptosis-dysregulated tumors.

We identify PLEC as a candidate functional contributor associated with aggressive PTC phenotypes through multi-level preliminary validation. PLEC overexpression correlated with aggressive clinicopathological features including nodal metastasis (*P* = 0.009), advanced histological variants (*P* < 0.001), and residual disease (*P* = 0.003) – independent of gender/age covariates. ROC analysis suggested preliminary diagnostic relevance of PLEC expression; however, these data do not establish diagnostic superiority or clinical applicability. Larger independent cohorts and multivariable analyses are required to determine whether PLEC has robust biomarker value in PTC. In our study, PLEC knockdown suppressed PTC cell proliferation, migration, and induced apoptosis. As a cytoskeletal linker protein, PLEC likely facilitates migration through integrin-cytoskeleton dysregulation, explaining its association with extracapsular extension – a novel mechanistic insight beyond its known roles in other carcinomas.

Our computational-to-experimental pipeline revealed six FDA-approved potential anti-tumor compounds (cidofovir, clodronate, fulvestrant, niclosamide, Troglitazone, and zoledronate). To our knowledge, Yang et al. (28) reported that cidofovir inhibited the proliferation of human papillomavirus-infected cervical cancer cells. Yang et al (29) reported that clodronate-induced apoptosis in human thyroid carcinoma is mediated, at least in part, through P2 receptor–dependent signaling. Kalinsky et al (30) further showed that fulvestrant combined with abemaciclib provides clinical benefit in advanced breast cancer following progression on prior CDK4/6 inhibitor therapy. In thyroid cancer models, Yu et al (31) demonstrated that niclosamide triggers apoptosis via the mitochondrial intrinsic pathway and suppresses migratory and invasive behavior *in vitro*. In addition, Muller et al (32) reported that the Troglitazone derivative EP13 disrupts cellular energy metabolism by inhibiting respiratory chain complex I, thereby enhancing the antiproliferative effects of glycolysis inhibitors in breast cancer cells. Desnoyers et al (33) suggest Zoledronate may have therapeutic relevance in selected breast cancer settings. In our data, Troglitazone is the compound with the lowest predicted binding energy for PLEC (-7.5 kcal/mol), forming stable hydrogen bonds with K190/S174/K143 residues. Such as MD simulations confirmed complex stability (RMSD <2Å after 100 ns). Crucially, rescue experiments suggested that PLEC may contribute to the cellular response to Troglitazone: PLEC overexpression reversed its anti-tumor effects across all assays (proliferation; apoptosis as well as migration). This specific antagonism distinguishes Troglitazone from non-selective kinase inhibitors and aligns with emerging evidence that thiazolidinediones exhibit *PLEC*-dependent activity in PTC.

While GEO validation strengthens subtyping generalizability, prospective validation in multi-center cohorts is warranted. The translational relevance of Troglitazone should be interpreted cautiously. Troglitazone is a thiazolidinedione and PPARγ agonist that was withdrawn from clinical use because of hepatotoxicity (34, 35). Therefore, the present findings should be viewed as proof-of-concept evidence supporting a PLEC-associated pharmacological vulnerability in PTC, rather than as support for immediate clinical repurposing of Troglitazone. Although our vivo experiment showed no overt hepatic or renal toxicity under the tested conditions, as indicated by ALT, AST, CRE, BUN, body weight, organ weight, and H&E staining, these short-term preclinical observations cannot fully address the known clinical hepatotoxicity risk of Troglitazone.

In addition, because Troglitazone is a PPARγ agonist (36), the observed anti-PTC effects cannot be attributed exclusively to PLEC-related mechanisms. PPARγ-dependent signaling, PLEC-associated cytoskeletal regulation, or cooperative effects between these pathways may contribute to the phenotype. Future studies should therefore investigate whether Troglitazone-induced anti-PTC effects are independent of, or coordinated with, PPARγ signaling (37). From a translational perspective, safer Troglitazone derivatives, structurally optimized analogs, or targeted delivery systems should be evaluated to reduce hepatotoxicity while preserving anti-tumor activity. Additional pharmacokinetic, pharmacodynamic, hepatotoxicity, and patient-derived model studies will be required before any clinical application can be considered.

Several limitations of this study should be acknowledged. First, the molecular subtyping and candidate-gene prioritization were derived primarily from retrospective TCGA and GEO datasets that differed in platform, sample composition, and clinicopathological annotation. Although selected findings were examined in external datasets, the stability and clinical relevance of the A1–A4 classification require prospective validation in larger, multicenter, and histologically well-defined PTC cohorts. Moreover, the subtype analysis was intended as an exploratory framework, and subtype-specific responses to Troglitazone were not directly evaluated. The immune-cell fractions estimated using CIBERSORT were computational inferences from bulk transcriptomic data and therefore require orthogonal validation. Second, validation using 15 paired clinical specimens supports only preliminary clinical associations, while the functional experiments were conducted mainly in B-CPAP and TPC-1 cells, which harbor established oncogenic alterations. Consequently, the present evidence supports PLEC as an aggressiveness-associated candidate and functional contributor rather than establishing it as an initiating oncogenic driver. Stable loss-of-function models, gain-of-function studies in non-malignant thyroid cells, broader cell-line panels, transcriptomic profiling, and patient-derived models are warranted. Third, molecular docking, molecular dynamics simulations, CETSA, and rescue experiments collectively support a putative PLEC-associated interaction and drug response but do not conclusively demonstrate direct and selective binding or exclude contributions from PPARγ-dependent and other parallel pathways. Finally, the short-term xenograft and toxicity observations cannot address pharmacokinetics, long-term safety, or the known hepatotoxicity that led to the clinical withdrawal of Troglitazone. Troglitazone should therefore be regarded as a proof-of-concept compound rather than a candidate for immediate clinical repurposing, and safer analogs or optimized delivery strategies require comprehensive pharmacokinetic, pharmacodynamic, and toxicological evaluation.

## 5 Conclusion

In conclusion, this study identifies PLEC as a candidate molecule associated with aggressive PTC phenotypes and provides preliminary evidence that Troglitazone may exert anti-PTC effects through a PLEC-associated mechanism. The docking, molecular dynamics, CETSA, and rescue data support a putative interaction between Troglitazone and PLEC, but additional biochemical validation is required to confirm direct target engagement. These findings provide proof-of-concept evidence for further investigation of PLEC-associated pharmacological vulnerability in PTC, but safer analogs, optimized delivery strategies, and additional mechanistic and toxicity validation are required before clinical translation can be considered.

## Data Availability

The original contributions presented in the study are included in the article/**Supplementary Material**. Further inquiries can be directed to the corresponding authors.

## Supplementary material

Supplementary Figure 1 The expressions of hub genes for PTC. **P* < 0.05; ****P* < 0.001.

Supplementary Figure 2 The anti-PTC effect of Troglitazone. **(A)** MTT assay revealed the IC_50_ of Troglitazone treatment PTC cells for 48hrs. **(B)** AO/EB staining was used to test the apoptosis rate when treatment with Troglitazone. **(C)** Colony formation assay was used to test the proliferation of PTC cells after treatment with Troglitazone. **(D)** Cell migration rate was measured by a Transwell assay. ****P* < 0.001 vs Control group.
