# Supplementary material for: The role of plectin as a druggable target in papillary thyroid carcinoma: therapeutic potential of troglitazone
Source: Front Immunol. 2026 Aug 26;17:1789251. doi: 10.3389/fimmu.2026.1789251 (PMC13553872; doi:10.3389/fimmu.2026.1789251)
Supplement: Supplementary file 1 [file Table1.docx]

**Supplementary Table S1. Public datasets information**

| **Dataset / accession** | **Full public series or downloaded records** | **Samples included in the current study** | **Input data status** |
| --- | --- | --- | --- |
| TCGA-THCA — TCGA-THCA (GDC) | 551 STAR-Counts records were reported as downloaded under the stated GDC filters in the current manuscript. | The current manuscript reports 551 RNA-seq records, 512 PTC cases with clinicopathological data, and—separately in the Figure 3 ROC analysis—512 PTC plus 59 non-tumor samples. | Processed gene-level count data downloaded from GDC; raw FASTQ files were not analyzed. |
| TCGA-THCA — TCGA-THCA (GDC) | 483 MAF records were reported as downloaded in the current manuscript. | 483 mutation profiles. | Processed masked somatic mutation MAF files downloaded from GDC. |
| TCGA-THCA — TCGA-THCA (GDC) | 991 masked copy-number segment records were reported as downloaded in the current manuscript. | 512 PTC patients were reported as eligible for clinicopathological association analyses. | Processed masked copy-number segment files downloaded from GDC. |
| TCGA-THCA — TCGA-THCA (GDC) | 551 number of clinical records downloaded before eligibility and missing-data filtering. | 512 PTC patients were reported as eligible for clinicopathological association analyses. | Clinical data downloaded from GDC/TCGA. |
| GEO — GSE104005 | The GEO series design describes 30 thyroid carcinomas and 6 non-neoplastic thyroid tissues; the RNA-expression subseries contains 34 samples. | 29 thyroid carcinoma and 5 non-neoplastic thyroid samples, as reported in the current Methods section. | Processed expression data and a non-normalized supplementary text file are publicly available. |
| GEO — GSE33630 | 105 arrays: 49 papillary thyroid carcinomas, 45 normal thyroid tissues, and 11 anaplastic thyroid carcinomas. | 49 of PTC and 45 normal thyroid samples retained for the analysis. | Raw CEL files and processed expression data are publicly available. |
| GEO — GSE60542 | 92 array records with a complex design including primary PTC, nodal/other metastatic lesions, normal thyroid and lymph-node controls, and replicate samples. The series design includes 28 primary PTC and 24 normal thyroid specimens before replicate/exclusion handling. | 24 of primary PTC and 11 normal thyroid samples retained | Raw and processed expression data are publicly available. |

Notes: All datasets were derived from Homo sapiens. “Full public series or downloaded records” describes the public accession or the number reported as downloaded, whereas “Samples included” must reflect the final analysis-specific sample manifest. TCGA and GEO cohorts were analyzed independently unless explicitly stated otherwise. Replace every bracketed field with verified information and convert blue text to black before submission.

**Supplementary Table S2. Dataset definitions and performance metrics for receiver operating characteristic (ROC) analyses.**

**A. Datasets, outcomes, and analytical roles**

| **Figure panel** | **Dataset / accession** | **Platform / data type** | **Analysis groups** | **Predictor(s)** | **Outcome definition** | **Performance summary** | **Analytical purpose and interpretation boundary** |
| --- | --- | --- | --- | --- | --- | --- | --- |
| Figure 2D | GEO: GSE104005 | GPL14951; Illumina HumanHT-12 WG-DASL V4.0 R2 expression beadchip; processed gene-expression data | Thyroid carcinoma, n = 29; non-neoplastic thyroid, n = 5 | BRAF, NRAS, PLEC, ARAP3, BSN, and AKT1 | Positive class: thyroid carcinoma; reference class: non-neoplastic thyroid | AUC: BRAF, 0.57; NRAS, 0.61; PLEC, 0.75; ARAP3, 0.77; BSN, 0.44; AKT1, 0.60 | Exploratory external-cohort assessment of marker discrimination. The carcinoma group contains mixed histological types; therefore, the result is not a PTC-specific validation. |
| Figure 3F | TCGA-THCA (GDC) | RNA sequencing; processed PLEC expression values used in the exported ROC analysis | PTC (tumor), n = 512; non-tumor thyroid, n = 59 | PLEC (ENSG00000178209.16) | Positive class: tumor; reference class: normal/non-tumor | AUC, 0.796 (95% CI, 0.745–0.847) | Exploratory tissue-level discrimination of PLEC expression in TCGA-THCA. This single-cohort analysis does not establish independent clinical diagnostic performance. |

**B. Detailed operating characteristics for PLEC in TCGA-THCA (Figure 3F)**

| **Metric** | **Value** | **Metric** | **Value** |
| --- | --- | --- | --- |
| AUC | 0.796 | 95% confidence interval | 0.745–0.847 |
| Optimal cutoff | 3.7586 | Cutoff criterion | Maximum Youden index |
| Sensitivity | 73.24% | Specificity | 74.58% |
| Accuracy | 73.38% | Youden index | 0.4782 |
| Positive predictive value | 96.15% | Negative predictive value | 24.31% |
| Classification counts | TP = 375; TN = 44 | Classification errors | FP = 15; FN = 137 |
| ROC direction | Positive; automatically determined by pROC | Software | R 4.2.1; pROC 1.18.0; ggplot2 3.4.4 |

Notes: AUC, area under the curve; CI, confidence interval; FN, false negative; FP, false positive; GDC, Genomic Data Commons; GEO, Gene Expression Omnibus; NPV, negative predictive value; PTC, papillary thyroid carcinoma; PPV, positive predictive value; TCGA-THCA, The Cancer Genome Atlas Thyroid Carcinoma cohort; TN, true negative; TP, true positive. The optimal cutoff in Figure 3F was selected by maximizing the Youden index. PPV and NPV are prevalence-dependent and should not be generalized to screening or clinical populations. Figure 2D presents marker-specific AUC values; detailed operating characteristics are provided for the PLEC ROC analysis in Figure 3F.
