# Supplementary material for: The role of plectin as a druggable target in papillary thyroid carcinoma: therapeutic potential of troglitazone
Source: Front Immunol. 2026 Aug 26;17:1789251. doi: 10.3389/fimmu.2026.1789251 (PMC13553872; doi:10.3389/fimmu.2026.1789251)
Supplement: Supplementary file 2 [file Table2.docx]

**Supplementary Table S2. Dataset definitions and performance metrics for receiver operating characteristic (ROC) analyses.**

**A. Datasets, outcomes, and analytical roles**

| **Figure panel** | **Dataset / accession** | **Platform / data type** | **Analysis groups** | **Predictor(s)** | **Outcome definition** | **Performance summary** | **Analytical purpose and interpretation boundary** |
| --- | --- | --- | --- | --- | --- | --- | --- |
| Figure 2D | GEO: GSE104005 | GPL14951; Illumina HumanHT-12 WG-DASL V4.0 R2 expression beadchip; processed gene-expression data | Thyroid carcinoma, n = 29; non-neoplastic thyroid, n = 5 | BRAF, NRAS, PLEC, ARAP3, BSN, and AKT1 | Positive class: thyroid carcinoma; reference class: non-neoplastic thyroid | AUC: BRAF, 0.57; NRAS, 0.61; PLEC, 0.75; ARAP3, 0.77; BSN, 0.44; AKT1, 0.60 | Exploratory external-cohort assessment of marker discrimination. The carcinoma group contains mixed histological types; therefore, the result is not a PTC-specific validation. |
| Figure 3F | TCGA-THCA (GDC) | RNA sequencing; processed PLEC expression values used in the exported ROC analysis | PTC (tumor), n = 512; non-tumor thyroid, n = 59 | PLEC (ENSG00000178209.16) | Positive class: tumor; reference class: normal/non-tumor | AUC, 0.796 (95% CI, 0.745–0.847) | Exploratory tissue-level discrimination of PLEC expression in TCGA-THCA. This single-cohort analysis does not establish independent clinical diagnostic performance. |

**B. Detailed operating characteristics for PLEC in TCGA-THCA (Figure 3F)**

| **Metric** | **Value** | **Metric** | **Value** |
| --- | --- | --- | --- |
| AUC | 0.796 | 95% confidence interval | 0.745–0.847 |
| Optimal cutoff | 3.7586 | Cutoff criterion | Maximum Youden index |
| Sensitivity | 73.24% | Specificity | 74.58% |
| Accuracy | 73.38% | Youden index | 0.4782 |
| Positive predictive value | 96.15% | Negative predictive value | 24.31% |
| Classification counts | TP = 375; TN = 44 | Classification errors | FP = 15; FN = 137 |
| ROC direction | Positive; automatically determined by pROC | Software | R 4.2.1; pROC 1.18.0; ggplot2 3.4.4 |

Notes: AUC, area under the curve; CI, confidence interval; FN, false negative; FP, false positive; GDC, Genomic Data Commons; GEO, Gene Expression Omnibus; NPV, negative predictive value; PTC, papillary thyroid carcinoma; PPV, positive predictive value; TCGA-THCA, The Cancer Genome Atlas Thyroid Carcinoma cohort; TN, true negative; TP, true positive. The optimal cutoff in Figure 3F was selected by maximizing the Youden index. PPV and NPV are prevalence-dependent and should not be generalized to screening or clinical populations. Figure 2D presents marker-specific AUC values; detailed operating characteristics are provided for the PLEC ROC analysis in Figure 3F.
